# Look at the future -perceptions of fertility counseling and decision-making among adolescents and their parents in the context of hematopoietic stem cell transplantation—experience of one major center for pediatric stem cell transplantation

**DOI:** 10.3389/fped.2023.1249558

**Published:** 2023-11-29

**Authors:** Anke Barnbrock, Fabienne Hamannt, Emilia Salzmann-Manrique, Theresa Rohm, Sandra Lange, Peter Bader, Andrea Jarisch

**Affiliations:** ^1^ Division for Stem Cell Transplantation and Immunology, Department for Children and Adolescents, University Hospital, Goethe University, Frankfurt, Germany; ^2^ Division for Pediatric Hematology and Oncology, Department for Children and Adolescents, University Hospital, Goethe University, Frankfurt, Germany; ^3^ Department for Children and Adolescents, Klinikum Frankfurt Höchst, Frankfurt, Germany

**Keywords:** fertility counseling, pediatric oncology, pediatric stem cell transplantation, perception of fertility counseling, decision making in fertility procedures

## Abstract

**Introduction:**

Increasing survival rates after hematopoietic stem cell transplantation (HSCT) in childhood should put focus on improving the quality of life as adults. An essential aspect is fertility and its preservation. In order to take advantage of the possibility of fertility preservation, fertility counseling should be provided to patients and their parents prior to gonadotoxic therapies.

**Methods:**

The aim of this survey was to analyze the impact of fertility counseling in pediatric stem cell transplantation in patients and their parents using questionnaires designed for the study questions. Fifty-one parents and 7 adolescent patients were interviewed between February 2019 and October 2021 about the counseling, their perceptions of fertility issues, and the nature of decision- making concerning fertility preservation. The study included patients with malignant (e.g., leukemia, lymphoma, neuroblastoma) and nonmalignant diseases (e.g., thalassemia, sickle cell disease, immunodeficiency) who received counseling on fertility preservation before HSCT based on an in-house standard and analysed the impact for both groups.

**Results:**

Two-thirds of the study participants were concerned about having children and grandchildren respectively; for half of all respondents, the topic of fertility and fertility preservation proved to be hopeful. Forty percent of the study participants were burdened by the risk of possible fertility limitations after HSCT. Concerns about fertility was particularly significant for parents whose children were advised to undergo fertility preservation. Parents of children <12 years found deciding on appropriate measures more difficult. Parents with children >7 years involved their children in the decision. All study participants agreed that fertility counseling had not negatively affected the parent-child relationship. More than 90% of all study participants were in favor of addressing fertility, its potential limitations and fertility preservation measures before HSCT. There was no significant difference between the malignant and the non-malignant cohort in all study questions.

**Discussion:**

Overall, the standardized fertility counseling provided in our center of pediatric stem cell transplantation resulted in high satisfaction among patients and their parents. Multiple counseling on infertility risk, including the younger patients in the decision-making and further options after gonadotoxic therapy may increase the satisfaction of the counseled patients and their parents.

## Introduction

Fertility is one of the essential capabilities of human beings both for the individual and for the continuity of society. Negatively influencing factors include vital therapies with a broad spectrum of side effects such as chemotherapies and radiation. Hematopoietic stem cell transplantation (HSCT) has a particularly high risk of fertility impairment as a therapy-related complication, at 75% (1).

In the treatment of children and adolescents, fertility and its preservation prior to gonadotoxic therapy is an issue of increasing relevance. About 80% survived 30 years after diagnosis of childhood oncologic disease (2). Similarly, the current 5-year survival rate for HSCT ranges from 65% to 93%, depending on the underlying disease and type of transplant (3, 4).

In our experience all information that parents receive during the pre-transplant interview, the risk of infertility appears to be one of the most difficult side effects to realise. Infertility means a disruption of life continuation because there will not be a next generation. Therefore, physicians have to offer various possibilities for FP that are adjusted to special needs and medical conditions of the individual patient and available medical resources. Therefore, a multidisciplinary counselling session, access to fertility preserving measures and an opportunity for their own family planning, is mandatory.

Fertility counseling before chemotherapy and other gonadotoxic treatments lowers the level of fertility-related anxiety in patients and is associated with better mental health (5, 6). Thus, it is essential in terms of patients' long-term psychological well-being to provide fertility counseling before gonadotoxic therapy.

The establishment of a uniform educational session on fertility issues after HSCT as well as counseling on possible fertility-preserving measures should be understood as a self-evident and obligatory part of the pre-transplant interview. A recent study shows that despite recommendations for fertility counseling overall only 39% of patients received counseling prior to HSCT (7). A similarly low rate was shown by other publications as well (8, 9).

Currently, fertility counseling and the implementation of fertility-preserving measures vary within Europe depending on national recommendations, technical expertise, local circumstances, and cost coverage by the health care system (10–12).

The questionable cost recovery for counseling and associated low staffing are reasons why not all pediatric departments handling gonadotoxic therapies have established fertility counseling to date. Different to some other countries there is no general coverage by the health insurance for fertility preserving measures.

The aim of the conducted study is a survey on the quality and impact of fertility counseling in pediatric HSCT by interviewing parents and patients >12 years about their handling of fertility issues, their decision-making, as well as an assessment of fertility counseling, both, for HSCT indication for non-malignant and a malignant diseases in patients.

## Participants, material and methods

In this monocenter, noninterventional study we included patients and parents who received fertility counseling at our center. This prospective study included all pediatric patients who received their first HSCT for malignant or non-malignant disease from February 2019 to October 2021. Attendance at previous fertility counseling appointments prior to first HSCT was not cause for exclusion. Patients who had already undergone multiple transplants and patients and their parents who could not answer the German questionnaires without a translator due to a language barrier were excluded from the study though off course they received a fertility counseling translated in mother-tongue as well.

In children <12 years, the parents were invited to participate in the survey and in adolescents 12–18 years, the patient and parents were invited to participate.

As a standard procedure, each patient (age-dependent) and his or her guardian received an individual 60-min counseling session by a pediatric oncologistof the HSCT team prior to HSCT. An in-house standard operating procedure (SOP) for fertility counseling implemented at our institution was used to facilitate and standardize counseling. This procedure was published in 2022 (10). Recommended procedures accouring to the inhouse standard included ovarian tissue and oocyte cryopreservation for the female patients (depending on age and disease) and sperm and prepubertal testicular tissue cryopreservation for male patients (depending on age).

The counseling sessions included the individual risk assessment of infertility, description of the recommended procedures, and individualized and rational decision- making with the patient and his or her guardian. Patients who were initially counseled in the oncology department and required HSCT during the course of therapy were counseled again.

The coded and anonymized questionnaire was administered to the parents and patients >12 years within the first 2 weeks of the inpatient stay in the context of the HSCT. This was done by an independent person to avoid that study participants answer in a socially desirable way.

Approval for the study was granted by the Ethics Committee of the University Hospital Frankfurt am Main (business number 86/18). The study was conducted according to the principles of Declaration of Helsinki.

### Structure of the questionnaires

Two versions of questionnaires were developed, one for parents and the other for patients >12 years. The study focused on three main questions: (1) Perception of fertility issues prior to HSCT, (2) Decision-making about fertility preserving measures, (3) Evaluation of the fertility counseling. For this purpose, the questionnaire had four parts. Part A contains questions about sociodemographic data; part B covered medical history and fertility counseling in the past. Some questions were directed at patients and their guardians who received fertility counseling prior to the current counseling as part of a previous therapy. This group was treated as a subpopulation. Part C addressed the perception of fertility in the context of treatment, the patient's desire to have children and the parent-child relationship. Part D included the decision-making for or against fertility-preserving measures. Last, Part E assessed the individual fertility counseling. The questionnaire included 54 and 53 questions in the version for parents and for patients >12 years, respectively.

To improve the questionnaires, a pilot study had been conducted in the department of oncology as part of a partner study including 12 adolescents and parents of children 0–18 years as well as a number of healthy adolescents (e.g., medical students, volunteers) to improve understanding and age-appropriate vocabulary of the questionnaire.

### Statistical analyses

The evaluation of the questionnaires and the data analysis were performed with IBM® SPSS® Statistics, version 28.0.0.0 and R (version 4.3.0; R Foundation for Statistical Computing, Vienna, Austria). Data from parents and adolescents were analyzed separately. Descriptive statistics for categorical data are presented as absolute frequencies and percentages. For metric data, the mean, median, dispersion measures, and interquartile ranges were calculated and minimum and maximum were reported.

To compare two populations that differed in the expression of an independent variable, the nonparametric Mann–Whitney *U* test was applied. Fischer's exact test was applied to test the independence of 2 nonmetric-scaled variables. An undirected relationship between 2 ordinal-scaled variables was tested by the Kendall-τ correlation coefficient and Spearman-rho correlation. To test the influence of an independent variable on a nominally scaled categorical variable, logistic regression analysis was used. A two-sided significance level of *α* = 0.05 was chosen for all statistical tests mentioned.

## Results

**Figure 1:**
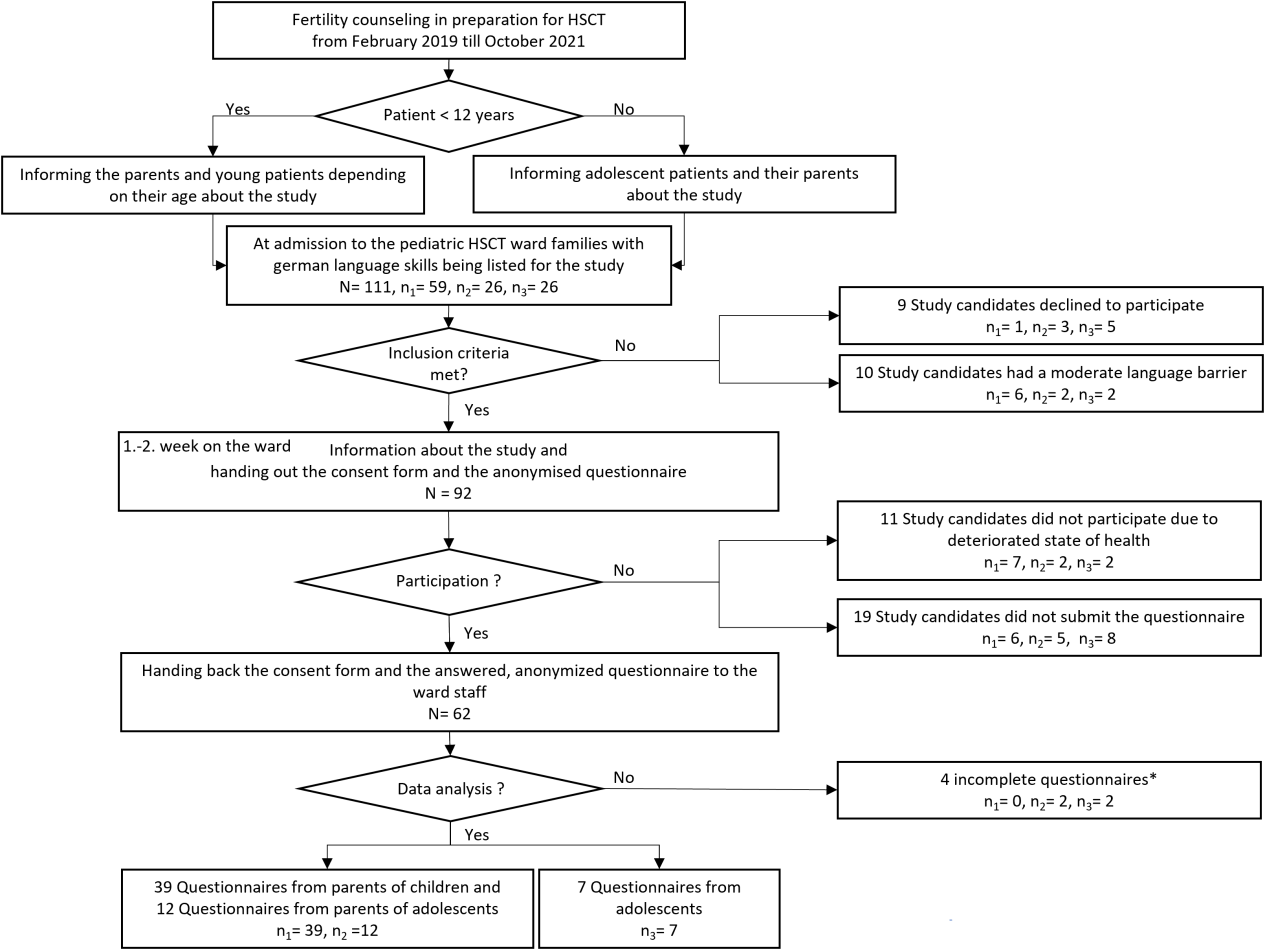
Flowchart of the study process. *Blank questionnaires and questionnaires with less than 30% responses were counted as incomplete. *N* represents the total population and results from the sum of *n*1, *n*2 und *n*3, *n*1 = parents of children; *n*2 = parents of adolescents; *n*3 = adolescents. There were 111 potential study participants temporarily listed. As shown in the flow chart a total of 92 participants were enrolled in the study and 58 questionnaires were analysed. How the number of analysed questionnaires derived from all participants is traceable by the dropouts in the right side of the flow chart.

A total of 92 participants were enrolled in the study. Figure 1 shows enrollment and exclusion of candidates due to language barrier, worsening health status or rejection of study participation. Fifty-three parents (response rate 72.6%) and 9 adolescents (response rate 47.4%) returned an edited questionnaire (overall response rate of 67.4%). Finally, 51 of 53 parent questionnaires were evaluable, 39 from parents with children <12 years and 12 from parents with adolescent children. Seven of 9 questionnaires of adolescents could be evaluated.

Supplementary Tables S1 and S2 shows the detailed distribution the demographic, socioeconomic, and clinical data in the study groups, divided into patients with malignant and non-malignant disease including 23 female and 28 male patients with a median age of 7.1 years (2 months–24 years).

Seventy-six percent of the patient cohort were German, 6% Turkish, 6% Syrian, 4% Afghan, and 8% other nationalities. Forty-three percent reported being Christians, 31% Muslims, 6% followers of other religion, 20% did not state a religious affiliation.

Forty-nine percent of the patient cohort had malignant disease, 51% a non-malignant disease. In the cohort of the no-malignant group there were 20 patients <12 years and 6 >12 years, in the malignant group 19 patients <12 years, 6 >12 years respectively.

**Figure 2:**
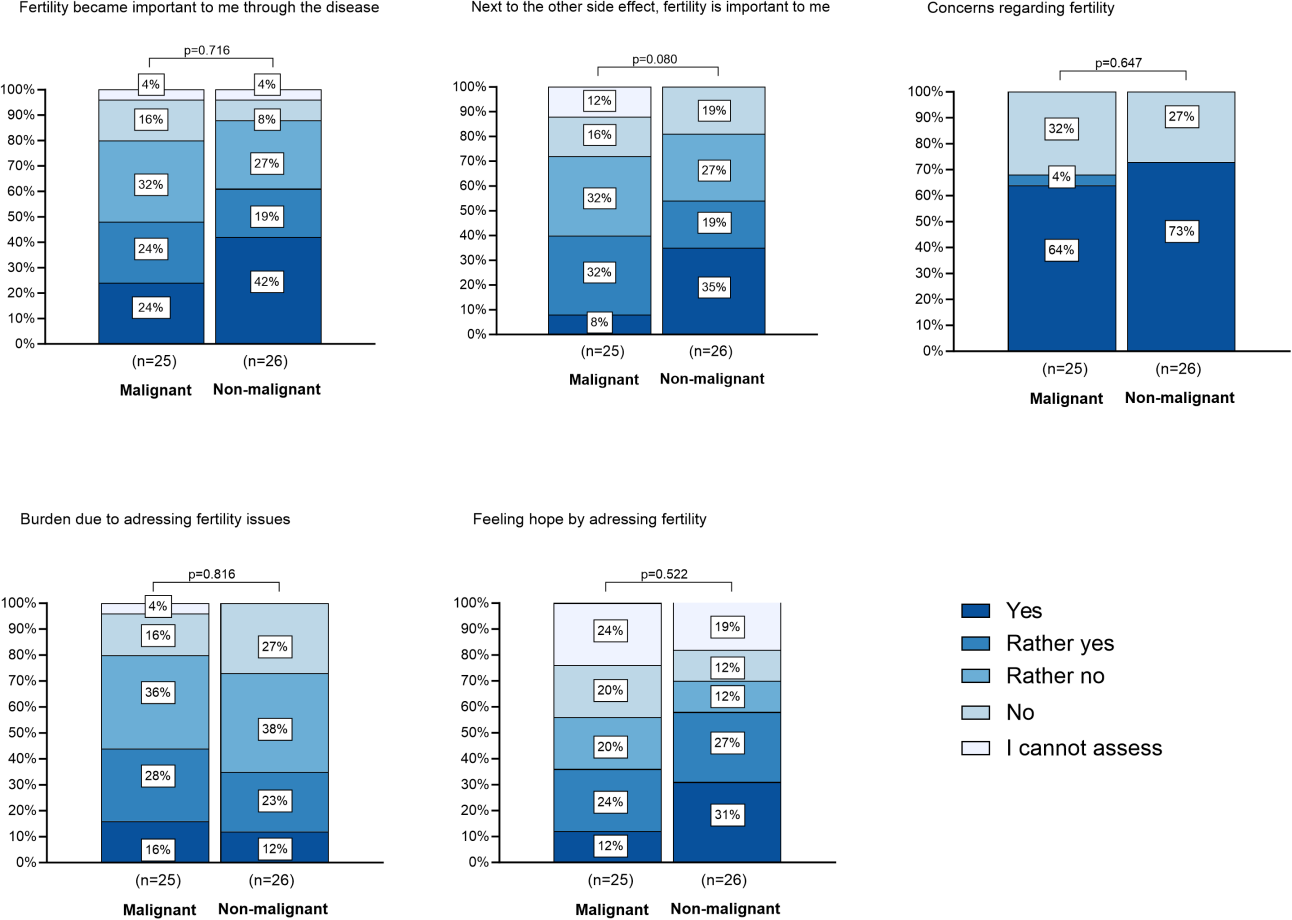
Impact of HSCT on perception of fertility. There are 5 barplots depicted. Each barplot shows the distribution of answers on selected questions to the impact of HSCT on perception of fertility. These questions were answered by the cohort of parents (*N* = 51). The participants are grouped by the malignancy of their child's disease. The legend shows the graded answers from “yes” to “I cannot assess”, which are also graded in color in the diagram. The height of each bar represents the relative frequency of the answer to each topic. The *p*-value on top of each barplot shows that there is no significant difference between the answers of parents of children with malignant and non-malignant disease.

Overall, 90.2% of parents were in favor of addressing fertility and fertility preservation before HSCT. Figure 2 presents the response behavior of the malignant and the non-malignant cohort to 5 questions on the perception of fertility. Concerns about child fertility were affirmed by most respondents regardless of the answer to the other questions. There was no significant difference in the perception between the malignant and the non-malignant cohort.

**Figure 3:**
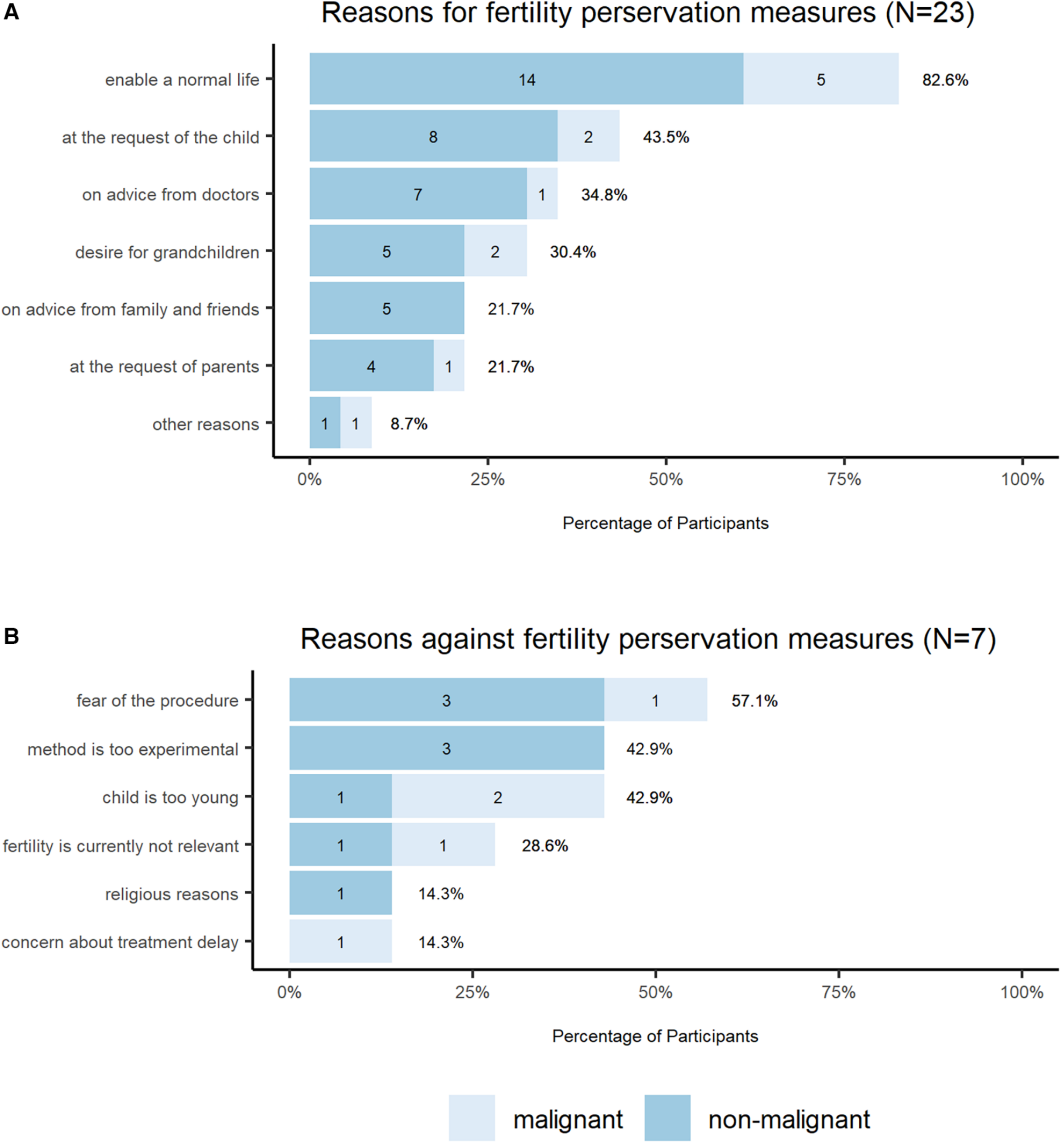
Reasons for fertility preservation decision. The bar charts show the (**A**) reasons for and (**B**) against fertility preservations measures, which were crucial for the decision-making. The total *N* of the participants who had the choice to take fertility preservation measures is *N* = 30, 23 (**A**) decided to take fertility preservation measures and 7 (**B**) decided against it. The response option included multiple choice. The legend shows two colors, which mark the answer of the parents of children with malignant and non-malignant disease.

For 82.6% of all parents (*n* = 30, distribution in non-malignant/malignant cohort is depicted in Figure 3), the main reason for performing the fertility preservation procedure was the desire to allow the child to live a normal life. Other reasons were the explicit wish of the child (43.5%), the advice of the doctors (34.8%), the desire to have grandchildren (30.4%), the wish of the parents (21.7%), the advice of family and friends (21.7%), to give the child the opportunity to make his or her own decision (4.3%), and to use the fertility preservation option (4.3%) (Figure 3A).

Concerns about the procedure was the most common reason for deciding not to undergo fertility preservation procedures (57.1% of the total cohort, *n* = 30). Further reasons were young age of the patient (42.9%), experimental character of the procedure (42.9%), and fertility was not currently a relevant issue (28.6%). Treatment delay and religious reasons were named in one case respectively (Figure 3B).

In 18 of the 30 families (60%) decision-making included the child, including 2 families who decided explicitly at the child's request. Eleven of 30 parents (37.9%) found it difficult to decide. Decision-making without involving the child proved to be independent of sociodemographic factors such as gender, religion, and nationality, as well as independent of the malignancy of the child's disease. The age of the child, on the other hand, correlated strongly with parental decision making. For children <7 years of age, the decision regarding fertility-preserving measures was usually made by parents alone, and children >7 years were included in the decision-making process (*p* = 0.005). There was no significant difference between the malignant and the non-malignant cohort.

Having their own children was important to 68.6% of parents of the total study population (*n* = 35, non-malignant cohort: *n* = 20, malignant cohort *n* = 15), 60.8% indicated that having their own children was important to their children as well (*n* = 31, non-malignant *n* = 19, malignant: *n* = 12). Fertility had become important to more than half of the patients due to the confrontation with this topic in the context of the disease (*n* = 28, non-malignant *n* = 16, malignant: *n* = 12). 39.2% of parents felt burdened by the issue of fertility (*n* = 20, non-malignant: *n* = 9, malignant: *n* = 11), 47.1% of parents saw fertility preservation as a source of hope (*n* = 24, non-malignant *n* = 15, malignant *n* = 9). In 68.6%, concerns and in 78.7%, doubts about HSCT as a therapy were not increased by fertility counseling. In 21.4% of parents concerns about HSCT were heightened, almost exclusively parents of children with non-malignant diseases (*n* = 8, non-malignant: *n* = 6, malignant: *n* = 2). However, this did not affect opinion about the necessity of HSCT in 86.3%. Fifty percent of parents considered fertility loss important among other side effects (*n* = 24, non-malignant *n* = 14, malignant: *n* = 10). According to the parents, the topic of fertility had not negatively influenced the parent-child relationship. The own relationship with the child was perceived as strengthened by 11.8% of the interviewed parents.

Forty-three of 49 families (87.8%) were satisfied with the counseling and recommended measures. 3 were rather not satisfied and another 3 could not assess their satisfaction.

For 44 of 49 parents, the counseling was understandable for themselves and their child, the duration was judged sufficient by 42 of 47 parents, 87.5% were able to ask their questions during counseling. Six parents who were unable to ask their questions named emotional overload (3 parents), the presence of the child (1 parent) and unspecific issues (2 parents). 17.8%, would have preferred counseling without a child.

61.7% had no suggestion for improvement of pre-HSCT fertility counseling. The most common suggestion for improvement (7 parents) was proving written information material, more appointments (5 parents), more time for counseling (4 parents) and more time on reflection (5 parents).

There were no significant differences between parents of patients with malignant and non-malignant disease on any variable, neither on the topic of burden of fertility (*p* = 0.231), satisfaction with interventions/counseling (*p* = 0.920), nor on importance of fertility as a source of hope (*p* = 0.375) or the weighting of fertility loss among other side effects of HSCT (*p* = 0.846). Within the whole study cohort here were no significant differences between parents of male and female patients on any variable.

The correlation of the parents' desire to have children and the assessment of the child's desire to have children showed a significant correlation (*p* = 0.022).

### Subgroup analysis: multiple counseled families

The subpopulation included 21 patients (11 malignant, 10 non-malignant disease) who received multiple fertility counseling, if the indication for HSCT arose in the course of therapy. Within this subgroup fertility-preserving measures were recommended in 13 cases (7 male, including 4 prepubertal and 3 post-pubertal measure, 6 female patients, 5 ovarian tissue, 1 oocyte cryopreservation). 68.4% of parents indicated that they had become more involved with the topic since the first consultation. Slightly more than half had talked to their own child about fertility since then, while 42.1% had not. The majority of respondents found it easier to understand the content of the survey during the second consultation. The second appointment was easier for the majority and 73.2% of the subpopulation were better able to follow and understand the 2nd consultation.

Opinion about fertility had not changed for 68.4% since first consultation, though familiarity with the counseling did not facilitate the decision-making for 72.6%.

**Figure 4:**
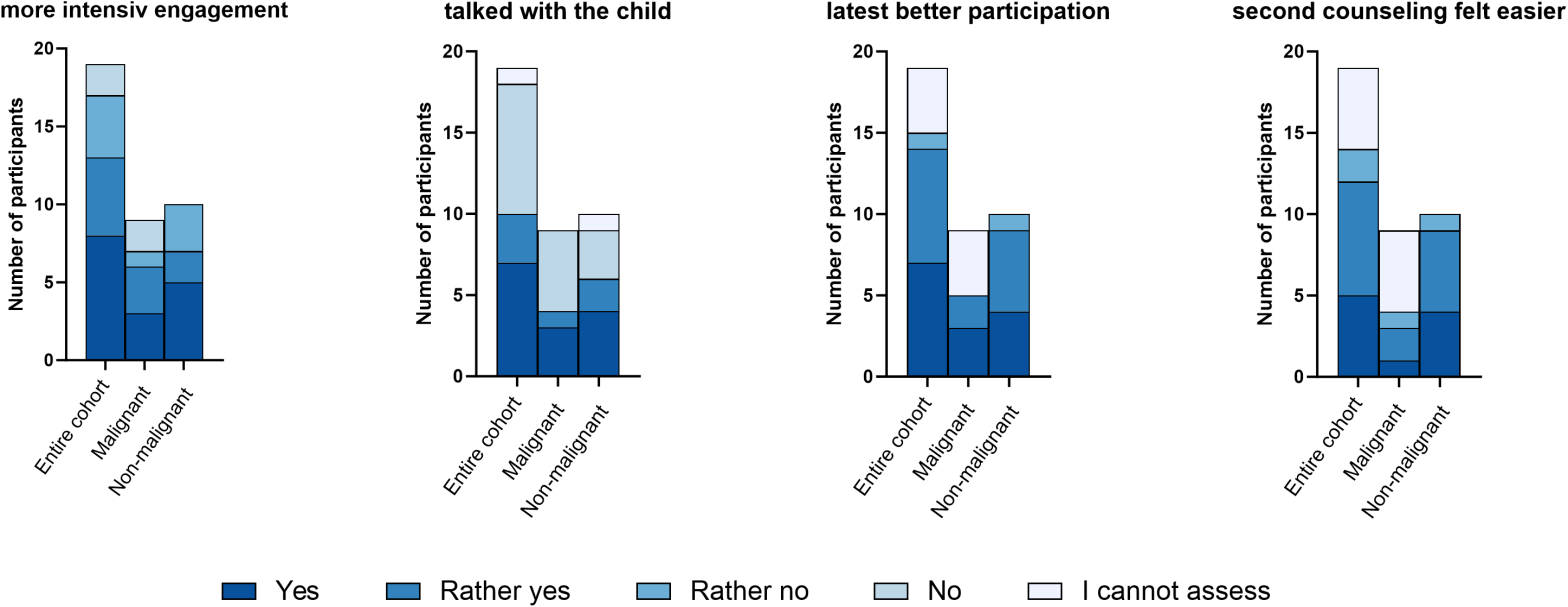
Impact of the first to the second consultation. 4 grouped bar plots present the answers to 4 questions how the first consultation impact the second. These questions were answered by 19 participants, who had more than one consultation. The legend shows the graded answers from “yes” to “I cannot assess”, which are also graded in color in the diagram. The height of each bar represents the relative frequency of the answer to each topic.

Figure 4 visualizes the response behavior of the subpopulation on the impact of the first to the second consultation for the entire cohort and the subgroups non-malignant/malignant cohort in terms of engagement in the topic, talking with the child, participation in and processing of the counseling.

**Figure 5:**
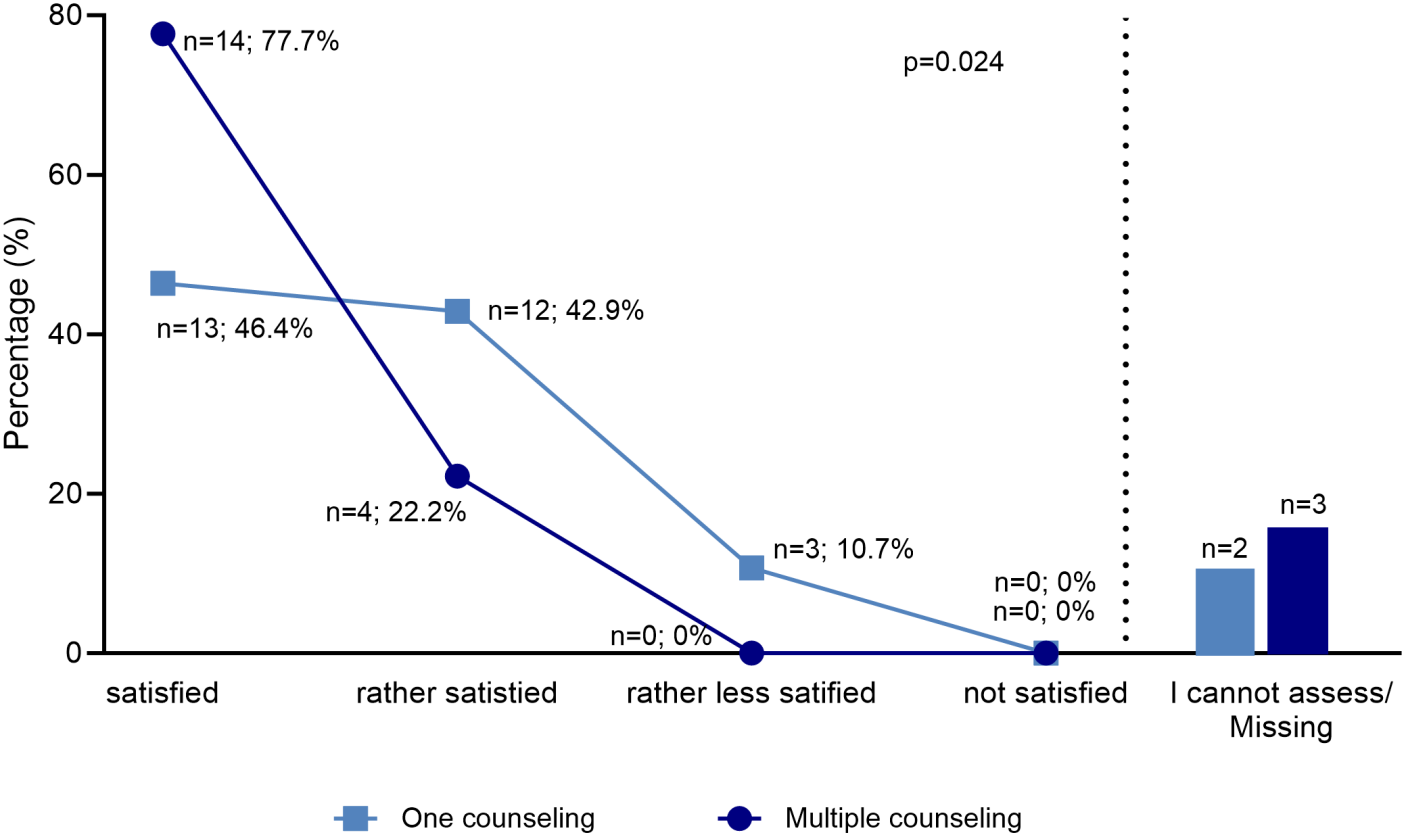
Satisfaction after one and multiple counseling. The diagram shows in two graphs the satisfaction of two groups, representing the subgroup of those who have been counseled several times and the group that has been counseled once. The legend shows two colors, which mark the two groups. The multiple counseled parents proven to be more satisfied. The difference between the groups was tested by Mann-Whitney-*U* with a significance level of *p* = 0.018.

For the subpopulation of multiple counseled families, there was a significant difference compared to the rest of the study population. Parents who had a history of fertility counseling were found to be more satisfied with the counseling and interventions than parents who were counseled for the first time in the context of HSCT (*p* = 0.018) (Figure 5). Furthermore, the group showed a tendency to interpret fertility preservation differently as a source of hope (*p* = 0.055). There was no significant difference in the other variables tested, burden of addressing fertility, fertility loss as an important side effect of HSCT, and decision severity.

### Results of the adolescents' survey

There were only 7 questionnaires of adolescents available, thus no statistical analysis was possible. All 7 patients were advised to seek fertility counseling by the oncology team or the HSCT team.

Four out of 7 patients had thought about their family planning before the consultation. 4 adolescents had concerns about their fertility because of HSCT.

Having their own children was important to 4 patients, 5 assumed that having grandchildren was important to their parents.

For 5 out of 7 adolescents, the topic of fertility had become more important at this stage of life due to the disease. For 5 patients, their fertility and fertility preservation was a hopeful factor during the disease phase. Four adolescents felt that the possible loss of fertility was important, in addition to other side effects of HSCT, 4 felt burdened by the issue of fertility. For 2 adolescents, concerns about HSCT had increased because of the fertility issue. Four adolescents found it easy to talk to confidants about the fertility issue. All patients denied that the topic of fertility affected their parent-child relationship. Six of 7 adolescents were in favor of fertility counseling.

Six of 7 patients were advised on fertility preservation measures, all opted for fertility-preserving measures when possible. The decision was made jointly with the parents by 4 patients. Three patients made the decision at their own request. One patient expressed that he made the decision at the request of his parents.

Six of 7 patients were satisfied with the counseling and felt that the counseling was understandable.

## Discussion

Fertility counseling prior to gonadotoxic treatments is highly relevant due to evolving therapies and improved survival rates. The current Society of Pediatric Oncology guideline mentions fertility preservation counseling, yet, contrary to adult medicine, there is no precise guideline for its implementation (13, 14). A survey from Diesch et al. aimed to analyze and compare different fertility preserving practices for children and adolescents across EBMT centers in 2013. A total of 177 pediatric centers reporting to the EBMT were contacted; 38 centers (21%) located in 16 different countries responded. Overall, 39% of the reported patients received counseling and 29% received a fertility preserving procedure. Therefore, increasing need for fertility preserving programs, for expanded training for the medical care teams, for more personal resources and funding has become imperative (7). Studies from other institutions have demonstrated that structured fertility counseling persuaded more patients to opt for fertility preservation measures (15). This reveals the urgent need for the standardization of fertility counseling, as also recommended by the Pediatric Diseases Working Party (PDWP) of the EBMT (16).

With this study we sought to analyze the meaning of the counseling for the adolescent patients and their parents and how the counseling is perceived. To our knowledge this is the first study performed in this immediate setting.

Considering the circumstances of just being admitted and starting therapy for HSCT and possibly the impression of the first therapy side effects such as infection or mucositis, which hinder or make participation impossible, our survey return rate is high and our study population represents the spectrum of pediatric HSCT very well though we had to exclude patients and families without secure German language skills due to the German questionnaire. In another setting it would be worthwhile to look for inter-cultural differences concerning the topic of fertility.

For half of the study participants, fertility with its potential limitations after HSCT represented a burden. The existence and extent of this burden in cancer patients is also shown in a variety of other studies (17–19). In our study no differences in burden could be found among different subgroups such as parents of patients with malignant and with non-malignant disease. For half of all respondents, the topic of fertility and possible fertility preservation proved to be a source of hope. Raising the issue of possible fertility loss and fertility preservation did not lead to doubts about the HSCT. It is striking that a similar distribution can be seen for the questions on the importance of fertility and burden of the issue. Neither did the counseling negatively affected the parent-child relationship. For some parents it actually strengthened the relationship.

Most of all respondents were satisfied with the counseling at our center, its comprehensibility and the recommended measures. Those who received counseling more than once had a significantly higher level of satisfaction. Sociodemographic differences and the type of disease of the child had no influence on the satisfaction of the parents. Similar results were found by von Wolff et al. in a survey regarding fertility concerns and counselling performance in an adult women cohort (20). The most common suggestion for improving counseling was a desire for more written information, more counseling appointments and longer time periods to reflect, and an offer of follow-up counseling. The desire for more information material concerning fertility preservation is also confirmed in other studies (15, 21, 22). Offers of a digital information platform that also provides decision support are particularly helpful (23, 24).

Parents of adolescent patients found it easier to decide on fertility preservation measures than parents of young children. Depending on the age of the children, the decision is usually made by the parents alone and place a great burden on them. Adolescents wish to be informed about the possible loss of fertility and want to be part in treatment-related decisions (25). As a consequence of the treatment due to an oncological disease, adolescents may develop fears about the loss of self-determination (26), the development of which can be prevented with integration into decision-making and counseling sessions (5, 6, 27). It emerged from the present study that parents of children >7 years were able to communicate with their children about their wishes for the future and make a decision together. A recent study showed that over 90% of boys >12 years were capable of participating in the decision process, whereas only 33% of children <12 years old did so. Other studies concluded that many adolescents and young adults felt overwhelmed with the decision to use fertility-preserving measures and advocated that they should decide together with their parents (19, 28, 29). Childrens' and adolescents' endorsement of their parents' participation is confirmed by the fact that until young adulthood, parents support the decision to preserve fertility in up to 80% of cases (25). In this context, the support of their family is more important to adolescents than the advice of physicians (30). However, studies discuss whether a higher dependence of oncological patients on their parents may lead parents to see themselves as the sole decision-makers, to underestimate their children's desire to have children, and to involve adolescents less in decision-making (25, 31).

In contrast to other studies the present work found no significant difference in distress concerning decision-making between parents of male and female patients (32). Others confirmed that both female and male patients are at high risk for severe burden (17).

The patient group with malignant vs. non-malignant diseases was differed in some aspects. Although there were no differences in burden, importance of fertility, decision severity, and satisfaction with counseling and interventions, there are differences concerning the pretreatment with potential gonadotoxic agents (33), risk of reimplantation of malignant cells (34–36) plus concerns like relapse and prognosis (21, 30, 37). Despite greater impairment of children with malignant disease, evidence suggests that they and their parents also want to be informed about fertility loss and counseled about their future prospects of having a family of their own (20, 38).

Parents counseled multiple times were shown to be more satisfied with both the counseling and the interventions even though it did not facilitate decision-making. A deeper understanding and good knowledge of medical interventions has been shown to lead to greater satisfaction and have a positive effect on mental health (5, 27).

Satisfaction is also related to one's active participation in counseling and decision-making concerning the procedures and leads to less doubt about the decision and thus contributes to higher quality of life (39). Less doubt about decision is most prevalent among patients who were able to ask questions during counseling (40). In the present study, 87.5% of parents expressed that they were able to ask their questions during counseling, which might result in the high score of satisfaction.

Satisfaction with one's decision is highest approximately one month after counseling (30). Nevertheless, follow-up at approximately 5 years after HSCT regarding satisfaction with measures and counseling would be useful for further understanding.

### Limitations

This is a single center study with only a small number of cases. The multinational composition of the patient population limited the number of patients who could be recruited for the German questionnaire due to the language barrier. The lower response rate among adolescents can be explained in part by the difficult circumstances surrounding the survey. Parents of severely ill patients are less likely to participate in surveys. In this view the setting of our study as to be critically reconsidered and weighed against the logistical advantages of a compact setting on the HSCT ward.

Thus, the survey results might only be a snapshot in the early phase of HSCT and might change with time. Poor or worsening health condition of the patients led to drop out of the study and in others may have influenced the response to the questionnaire and resulted in a lower response rate. Due to the very specific question, no validated questionnaire could be used. Larger and multicenter study trials are necessary to confirm our results especially as we had to rely on the German speaking study population only.

## Conclusion

It is desirable that every clinic treating children and adolescents with gonadotoxic therapies should also apply an SOP for counseling in clinical practice (7, 10). This SOP should be based on national (13, 41, 42) or international guidelines (16). In a published study in pediatric HSCT patients we were able to demonstrate the practicability of such an approach and the advantages of a standardized counselling concept in our clinic in a previous study (10).

Fertility counseling proves to be important to patients and families and results favor multiple counseling and the involvement in the decision-making process for pediatric patients even of young age, both for patients with malignant as well as non-malignant diseases and their parents.

## Data Availability

The original contributions presented in the study are included in the article/**Supplementary Material**, further inquiries can be directed to the corresponding author.
