# Supplementary material for: Look at the future -perceptions of fertility counseling and decision-making among adolescents and their parents in the context of hematopoietic stem cell transplantation—experience of one major center for pediatric stem cell transplantation
Source: Front Pediatr. 2023 Nov 29;11:1249558. doi: 10.3389/fped.2023.1249558 (PMC10716475; doi:10.3389/fped.2023.1249558)
Supplement: Supplementary file 1 [file Table1.pdf]

Suppl. Table 1: sociodemographic data of the study population by parent- age group’s patient and disease

	Total parent	Parent from patient < 12 y		Parent from patient ≥12 y		Patient ≥ 12 y	
		Non-malignant	Malignant	Non-malignant	Malignant	Non-malignant	Malignant
	N=51	N=20	N=19	N=6	N=6	N=4	N=3
**Questionnaire answered by, N (%)**							
Mother	36 (71)	11	17	4	4		
Father	13 (25)	7	2	2	2		
Other caregiver	2 (4)	2	0	0	0		
**Patient age**							
Median	7.1 y	4.8 y	6.8 y	15 y	14.5 y	15 y	15 y
range	2 m– 24 y	2m-11y	13m-11y	12y-24y	13y-15y	12y-24y	12y-15y
**Patient sex, N (%)**							
Female	23 (45)	8	10	4	1	2	0
Male	28 (55)	12	9	2	5	2	3
**Nationality, N (%)**							
German	39 (76)	16	14	4	5	3	3
Turkish	3 (6)	1	2	0	0	0	0
Syrian	3 (6)	2	0	1	0	1	0
Afghan	2 (4)	0	1	0	1	0	0
Other nationality	4 (8)	1	2	1	0	0	0
**Religion, N (%)**							
No religion	10 (20)	7	2	0	1	0	1
Catholic	14 (27)	2	7	2	3	2	2
Evangelic	8 (16)	2	5	0	1	0	0
Muslim	16 (31)	9	4	2	1	2	0
Other religion	3 (6)	0	1	2	0	0	
**Marital status of the parents, N (%)**							
Married	35 (69)	13	15	5	2	3	1
In a partnership	6 (12)	4	2	0	0	0	0
Divorced	2 (4)	1	1	0	2	0	1
Single / single parent	6 (12)	2	1	1	2	1	1
**Siblings, N (%)**							
None	8 (16)	6	2	0	0	4	3
At least one sibling	43 (84)	14	17	6	6	0	0
**Level of education, N (%)**							
No educational degree	6 (12)	2	2	2	0	0	0
School degree / Gesamtschule	4 (8)	1	2	1	0	2	0
Apprenticeship / Realschule	19 (37)	10	7	0	2	1	1
University degree / Gymnasium	20 (39)	7	7	3	3	1	2
Other	2 (4)	0	1	0	1	0	0
**Fertility counseling, N (%)**							
First time	30 (59)	13	10	3	4	3	2
Multiple fertility counseling	21 (41)	7	9	3	2	1	0

Abbr. y= years, m=months. Gesamtschule=Comprehensive secondary school, Realschule= Intermediate secondyra school, Gymnasium= High school.

Suppl.Table 2: clinical data of the patients by age and disease

	Total	**Patient < 12 y**		**Patient ≥12 y**	
		Non-malignant	Malignant	Non-malignant	Malignant
	N=51	N=20	N=19	N=6	N=6
**Patient's disease, N (%)**					
Leukemia	17 (33)	0	13	0	4
Myelodysplastic syndrome	4 (7)	0	3	0	1
Lymphoma	2 (4)	0	2	0	0
Neuroblastoma	1 (2)	0	0	0	1
Brain tumor	1 (2)	0	1	0	0
Immunodeficiency	11 (21)	10	0	1	0
Thalassemia	7 (14)	5	0	2	0
Sickle cell disease	4 (7)	1	0	3	0
Aplastic Anemia	4 (7)	4	0	0	0
**Past therapies, N (%)**					
Chemotherapy	25 (49)	0	19	0	6
Radiotherapy	2 (4)	0	1	0	1
Operation	5 (10)	2	1	1	1
Antibody therapy	11 (21)	3	8	0	1
Blood transfusion	3 (6)	0	0	3	0
MIBG therapy	1 (2)	0	0	0	1
Immunosuppressive and Immunoglobulins	1 (2)	0	0	1	0

Abbr. MIBG=meta-iodobenzylguanidine
