# Supplementary figures and images for: Look at the future -perceptions of fertility counseling and decision-making among adolescents and their parents in the context of hematopoietic stem cell transplantation—experience of one major center for pediatric stem cell transplantation
Source: Front Pediatr. 2023 Nov 29;11:1249558. doi: 10.3389/fped.2023.1249558 (PMC10716475; doi:10.3389/fped.2023.1249558)

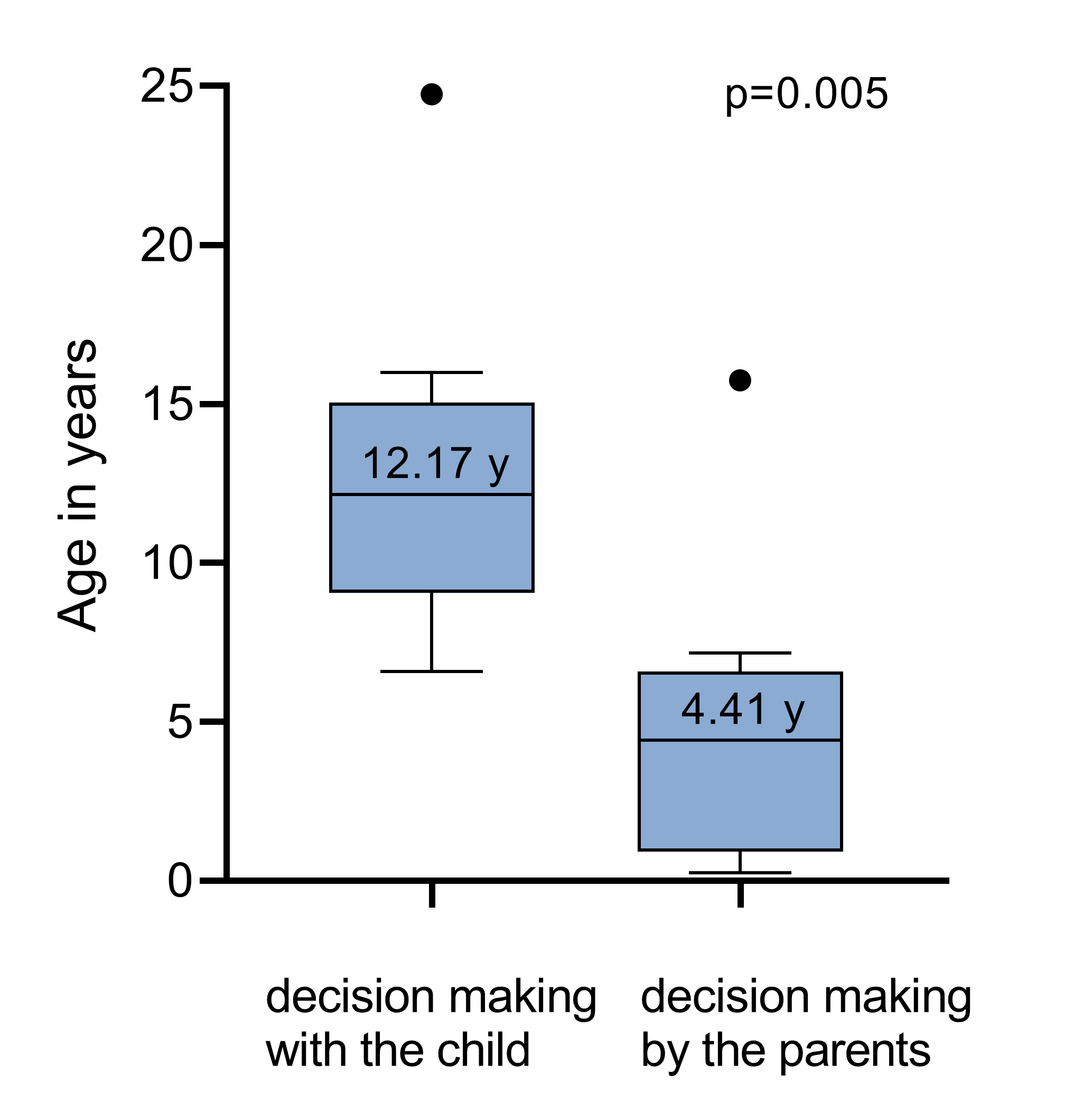

Supplement: Supplementary file 2 [file Image1.tif]

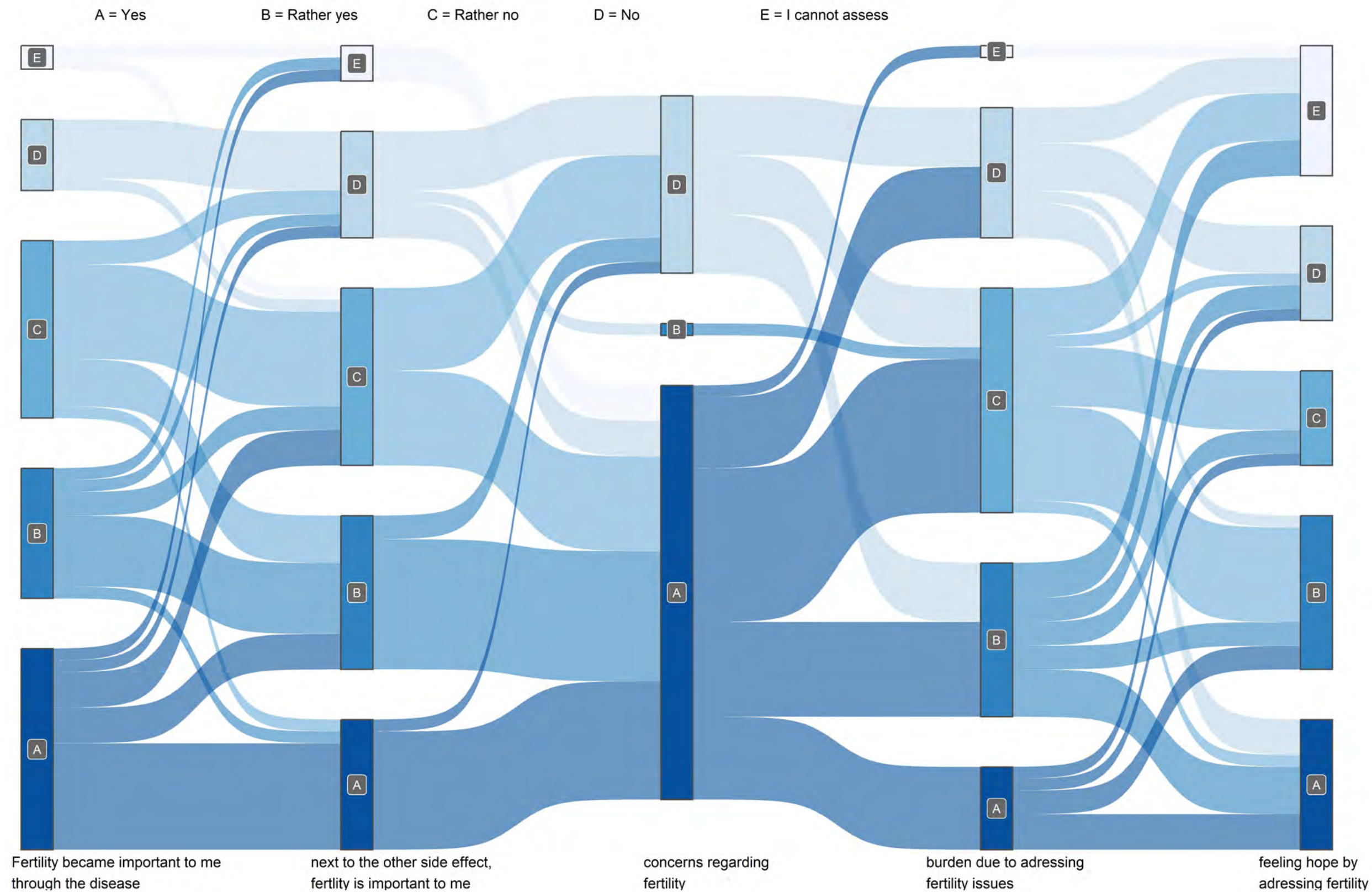

Supplement: Supplementary file 3 [file Image2.pdf]

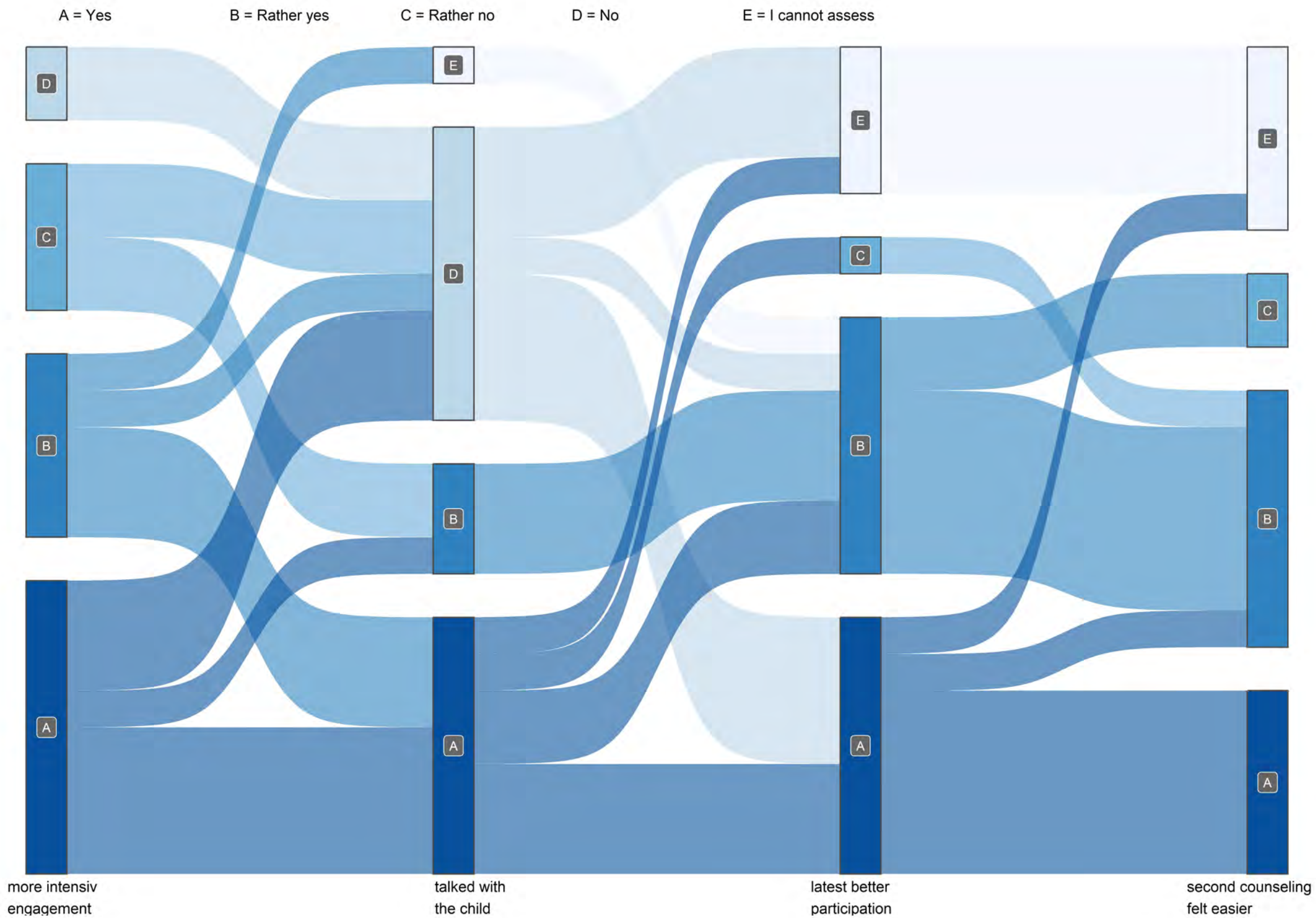

Supplement: Supplementary file 4 [file Image3.pdf]
